# Dolomite-Foamed Bioactive Silicate Scaffolds for Bone Tissue Repair

**DOI:** 10.3390/ma13030628

**Published:** 2020-01-31

**Authors:** Elisa Fiume, Dilshat Tulyaganov, Graziano Ubertalli, Enrica Verné, Francesco Baino

**Affiliations:** 1 Department of Applied Science and Technology, Politecnico di Torino, 10129 Turin, Italy; elisa.fiume@polito.it (E.F.); graziano.ubertalli@polito.it (G.U.); enrica.verne@polito.it (E.V.); 2 Department of Mechanical and Aerospace Engineering, Politencico di Torino, 10129 Turin, Italy; 3 Interdepartmental Center PoliTO BIOMedLab, Politecnico di Torino, 10129 Turin, Italy; 4 Interdepartmental Center J-Tech@PoliTO, Politecnico di Torino, 10129 Turin, Italy; 5 Department of Natural-Mathematical Sciences, Turin Polytechnic University in Tashkent, Tashkent 100095, Uzbekistan; tulyaganovdilshat@gmail.com

**Keywords:** scaffold, bioactive glass, glass–ceramic, biomaterials, bioceramics, porosity, bioactivity, bone tissue engineering, foaming, sustainable materials

## Abstract

The use of three-dimensional (3D) scaffolds is recognized worldwide as a valuable biomedical approach for promoting tissue regeneration in critical-size bone defects. Over the last 50 years, bioactive glasses have been intensively investigated in a wide range of different clinical applications, from orthopedics to soft tissue healing. Bioactive glasses exhibit the unique capability to chemically bond to the host tissue and, furthermore, their processing versatility makes them very appealing due to the availability of different manufacturing techniques for the production of porous and interconnected synthetic bone grafts able to support new tissue growth over the whole duration of the treatment. As a novel contribution to the broad field of scaffold manufacturing, we report here an effective and relatively easy method to produce silicate glass-derived scaffolds by using, for the first time in the biomedical field, dolomite powder as a foaming agent for the formation of 3D bone-like porous structures. Morphological/structural features, crystallization behavior, and in vitro bioactivity in a simulated body fluid (SBF) were investigated. All the tested scaffolds were found to fulfil the minimum requirements that a scaffold for osseous repair should exhibit, including porosity (65–83 vol.%) and compressive strength (1.3–3.9 MPa) comparable to those of cancellous bone, as well as hydroxyapatite-forming ability (bioactivity). This study proves the suitability of a dolomite-foaming method for the production of potentially suitable bone grafts based on bioactive glass systems.

## 1. Introduction

Implantation of three-dimensional (3D) porous scaffolds mimicking the trabecular architecture of cancellous bone is an excellent strategy to restore small- to mid-size defects of the osseous tissue due to fracture, bone resection surgery, or congenital diseases [1]. Using manmade biomaterials for making scaffolds is an advantageous approach that allows overcoming the limitations of transplant tissues (i.e., bone autograft, allograft, and xenograft), such as material shortage, risk of disease transmission from the donor to the patient, unpredictable resorption rate, and ethical/religious concerns [2,3].

Some special glass compositions exhibit the exceptional capability to bond to both bone and soft tissues, creating a stable interface and also promoting cell viability, healthy tissue regeneration and angiogenesis [4,5,6]. Therefore, bioactive glasses have been widely investigated over the last decades and are now clinically used in the form of fine powder and granules for filling osseous defects in orthopedics and dentistry, composites with polyethylene in ocular surgery (porous orbital implants), and fiber mats for wound healing in veterinary applications [7,8].

Given the versatility of glass processing, bioactive glasses have also shown great promise for making 3D porous scaffolds according to a number of fabrication methods. The research group led by Larry Hench, the inventor of the 45S5 glass composition (45SiO_2_-24.5CaO-24.5Na_2_O-6P_2_O_5_ wt.%) [9], first proposed the foaming of sols by using surfactants as a relatively easy approach to produce porous scaffolds based on gel-derived bioactive glasses [10,11,12]. Although some of these porous materials (e.g., the binary glass 70S30C (70SiO_2_-30CaO, mol.%)) successfully reached in vivo experimentation (rat tibial model) and proved to actually stimulate bone regeneration [13], sol–gel scaffolds typically suffer from high brittleness; furthermore, the sol–gel fabrication method requires a careful control on process parameters such as temperature and pH to ensure reproducibility and, potentially, industrial scalability of the products [14].

Another strategy was independently pioneered in 2006 by Park et al. [15] and Chen et al. [16] who applied the sponge replica method to process melt-derived powders of CaO-CaF_2_-P_2_O_5_-MgO-ZnO glass and 45S5 Bioglass^®^, respectively, using a commercial polyurethane foam as a sacrificial template. In both cases, the scaffolds exhibited a trabecular pore/strut architecture mimicking that of cancellous bone but the compressive strength was inadequate when compared to the typical range of human bone tissue (2–12 MPa [17]). Since then, this method became very popular to obtain bone-like porous ceramics and glasses due to its relative easiness and inexpensiveness. Optimization of basic glass composition and/or process parameters allowed producing bioactive glass and glass–ceramic scaffolds with high compressive strength (up to 18 MPa [18,19]), making them suitable for potential use in load-bearing bone sites, too.

Variants of this fabrication method include the use of natural or waste materials as porous templates, such as marine sponges [20] and stale bread [21] that were soaked in the glass powder suspension. If the porous template is immersed into a sol, hierarchical gel-derived scaffolds with multiscale macro-mesoporosity can be produced; for this purpose, not only the conventional open-cell polyurethane sponge [22] but also more unusual natural templates such as mushroom stalk [23] and cattail stem [24] have been experimented.

However, if high mechanical properties of the scaffold are required, space holder methods are usually preferred. In this technique, temporary material (i.e., the space holder) is mixed with the glass particles and devised as a sacrificial pore former for scaffolds. The use of polyethylene particles [25] and rice husk [26] allow obtaining strong scaffolds that are potentially suitable for use in high-load-bearing defect sites or even for the replacement of cortical bone; on the other hand, these porous implants typically suffer from low pore interconnectivity, which limits the permeation of biological fluids, cell colonization, and vascularization with obvious impairment of osteointegration.

Implementation of additive manufacturing techniques (AMTs) has recently opened new horizons in the fabrication of bioactive glass and ceramic scaffolds [27]. AMTs allow accurate control, tailoring, and reproducibility of scaffold features and pore/strut architecture, as well as an easy scalability to the industrial level [28]. Albeit the equipment for selective laser sintering [29] and stereolithography [30] still requires high investment costs, relatively affordable 3D printers are available on the market and can be customized on demand or in-house [31]. Furthermore, different materials (e.g., glass and polymers [32]) can be simultaneously processed by using 3D printing methods, thus obtaining composite scaffolds with finely tunable physico-chemical properties.

In most cases, however, scaffolds produced by AMTs exhibit a grid-like arrangement of macro-channels that do not properly mimic the trabecular architecture of cancellous bone [33,34]; on the contrary, sponge replication or foaming methods allow researchers to obtain bone-like porous structures.

In the present work, we propose a new method to produce foam-like silicate scaffolds based on a melt-derived SiO_2_-Na_2_O-K_2_O-MgO-CaO-P_2_O_5_ glass by using dolomite as a foaming agent. Dolomite was proved suitable as a foaming agent to fabricate porous glasses from waste materials [35]; however, to the best of our knowledge, its use was not reported so far for the fabrication of biomedical scaffolds. The foaming process involves the thermal decomposition of dolomite associated with CO_2_ production while small amounts of additional calcium and magnesium are incorporated in the glass-derived foam. In this regard, the choice of dolomite as a foaming agent is appropriate for the intended application as Ca^2+^ and Mg^2+^ ions are physiologically involved in bone metabolism and, when released from bioactive glasses, are known to stimulate osteoblasts towards a path of regeneration and self-repair [36].

## 2. Materials and Methods

### 2.1. Glass Production

The silicate glass used for scaffold fabrication is called 47.5B (47.5SiO_2_-10Na_2_O-10K_2_O-10MgO-20CaO-2.5P_2_O_5_ mol.%) [37] and was already reported to be suitable to make robocast porous structures for biomedical applications due to its favourable sintering behaviour and bioactive properties [38]. The glass was obtained by melting a homogeneous mix of the powdered precursors (SiO_2_, Na_2_CO_3_, K_2_CO_3_, (MgCO_3_)_4_·Mg(OH)_2_·5H_2_O, CaCO_3_ and Ca_3_(PO_4_)_2_, Sigma-Aldrich, St. Louis, MO, USA) up to 1500 °C in a platinum crucible. The glass was cast in water to obtain a “frit” and then milled by a zirconia ball miller (Pulverisette 0, Frtisch, Idar-Oberstein, Germany). Finer glass powders were eventually obtained after sieving (stainless steel sieve, Giuliani Technologies Srl, Turin, Italy; mesh 32 μm) and then used for further processing.

### 2.2. Scaffold Fabrication

Porous scaffolds were fabricated by using 47.5B glass as the main precursor and commercial dolomite from Dehkanabad deposit (Uzbekistan) (chemical composition (wt.%): 30.02 CaO, 19.63 MgO, 2.74 SiO_2_, 0.39 Al_2_O_3_, 0.27 Na_2_O, 0.10 K_2_O, 0.15 P_2_O_5_, 0.39 SO_3_, 0.01 MnO, 45.40 CO_2_, 0.90 others) as the foaming agent. In order to choose an appropriate content of the ingredients in the batch and decide on processing parameters, we referred to a previous study [35] that dealt with the foams based on sheet glass/fly ash composition with up to 5 wt.% of dolomite addition. Thus, considering the fact that appreciable foaming efficiency might be achieved at relatively low temperatures (e.g., 800 °C) with just 2 wt.% of dolomite incorporation [35], the formulation in the current investigation was decided to include 98 wt.% of 47.5B glass powder and 2 wt.% of dolomite (Table 1). Experimental batches were prepared by dry mixing glass and dolomite particles in a planetary mill for 30 min. The thoroughly mixed batches were placed into cylindrical stainless steel molds with a diameter and height of 10 mm and 15 mm, respectively. Heat treatment was undertaken in an electrically heated furnace (Carbolite type 3216 box furnace, Carbolite, Sheffield, UK) in air at 800 °C and 850 °C for 30 min (heating rate of 5 °C min^−1^); the obtained samples were referred to as D-800 and D-850 scaffolds, respectively. After cooling to room temperature, the scaffolds were removed from the molds and subjected to further investigations.

### 2.3. Characterizations

The distribution of glass particle sizes (vol.% vs. particle diameter) was assessed by a powder size analyzer (LS230, Beckam Coulter Corporation, Indianapolis, IN, USA).

The porous scaffolds were investigated from morphological and microstructural viewpoints. Field-emission scanning electron microscopy (FE-SEM) images were acquired (FE-SEM Supra^TM^ 40, Zeiss, Oberkochen, Germany) in order to assess the outcome of the foaming and sintering processes in terms of size of the structural features (pores and struts) and particle consolidation. The samples were sputter-coated with chromium before undergoing FE-SEM analysis and inspected at a voltage of 15 kV.

Crystallization of 47.5B scaffolds due to thermal treatment was investigated by X-ray diffraction (XRD; X’Pert Pro PW3040/60 diffractometer, PANalytical, Eindhoven, Netherlands). The analysis was performed varying the 2θ angle from 10° to 70°; the voltage was set at 40 kV and the filament current at 30 mA. Bragg–Brentano camera geometry was used, including Cu Kα incident radiation (λ = 0.15405 nm). Data were acquired fixing the step counting time at 1 s and the step size at 0.02°. The scaffold was ground into powder prior to undergoing XRD investigation. Identification of crystalline phases was carried out by using X’Pert HighScore software 2.2b (PANalytical, Eindhoven, The Netherlands) equipped with the PCPDFWIN database.

The total porosity P (vol.%) of the scaffolds was assessed in quintuplicate by density measurements through the calculation of the mass-to-volume ratio (*ρ*: apparent density; *ρ*_0_: bulk density) [39]:

(1) $$P=\left(1-\frac{\rho }{\rho_{0}}\right)\times 100$$

The samples were mechanically tested under compressive loads (MTS Model 43, MTS, Eden Prairie, MN, USA; cell load 5 kN; cross-head speed 1 mm/min). The compressive strength σ_c_ (MPa) was calculated as the ratio between the maximal load observed L_M_ (N) and the resistant cross-section A_r_ (mm^2^), which was measured for each sample by using digital calipers:

(2) $$\sigma_{c}=\frac{L_{M}}{A_{r}}.$$

The results were expressed as mean ± standard deviation assessed on five specimens, which were polished prior to the test by using SiC grit paper.

The bioactive properties of scaffolds were assessed upon soaking in a simulated body fluid (SBF), which was prepared according to the protocol reported by Kokubo and Takadama [40]. In vitro bioactivity tests were performed by immersing the scaffolds in SBF at 37 °C up to 7 days in static conditions. A mass-to-volume ratio of 1.5 mg/mL was used, as suggested in a previous study by the Technical Committee 4 (TC04) of the International Commission on Glass (ICG) [41]. The solution was completely replaced with fresh SBF every 48 h in order to simulate fluid circulation in physiological conditions and the pH was daily monitored by using a digital pH-meter. At the end of the experiment, the samples were gently rinsed with distilled water, left to dry overnight at 37 °C in an incubator and stored in a sealed plastic box before undergoing morphological and compositional investigation (FE-SEM equipped with energy dispersive spectroscopy (EDS)).

## 3. Results and Discussion

**Figure 1:**
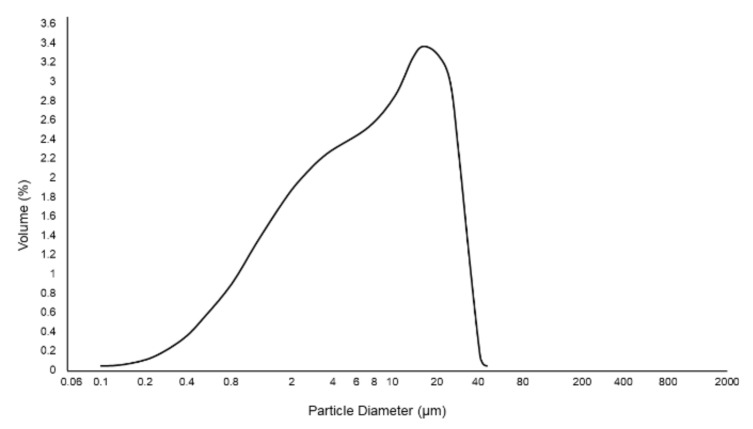
Particle size distribution of 47.5B glass powder (sieved below 32 μm) used for scaffold manufacturing.

The particle size distribution of glass powder used in this work is shown in Figure 1; mean particle size and specific surface area of glass and dolomite powders are reported in Table 1.

Glass-based scaffolds were produced for the first time by using dolomite powders (CaMg(CO_3_)_2_) as a foaming agent to obtain a porous and interconnected 3D structure for potential bone replacement in small- to mid-size bone defects.

The open-cell architecture obtained in this study was the result of the formation of gaseous CO_2_ upon sintering at 800 °C and 850 °C, which is one of the reaction products of the thermal decomposition of CaMg(CO_3_)_2_.

Table 1 summarizes the properties of the precursors used for the preparation of the 47.5B-based foams.

In previous studies, DTA analysis performed on CaMg(CO_3_)_2_ revealed two endothermic peaks, located at 800 °C and 890 °C and associated, respectively, to the decomposition of magnesium carbonate (Equation (3)) and calcium carbonate (Equation (4)) [35,42]:

(3) $$\mathrm{CaMg}{\left({\mathrm{CO}}_{3}\right)}_{2\;}↔{\mathrm{CaCO}}_{3}+\mathrm{MgO}+{\mathrm{CO}}_{2}$$

(4) $${\mathrm{CaCO}}_{3}↔\mathrm{CaO}+{\mathrm{CO}}_{2}.$$

The final reaction products deriving from the thermal decomposition of CaMg(CO_3_)_2_—i.e., CaO, MgO and CO_2_—were found to play an important role both in the definition of the compositional features and in the formation of the porous structure of the scaffolds: in fact, while CaO and MgO entered the glass network, gaseous CO_2_ could act as a foaming agent.

It is worth pointing out that, from a biological point of view, both Ca and Mg are strongly beneficial for bone regeneration. Specifically, Ca is known to increase osteoblast proliferation, differentiation, and extracellular matrix (ECM) mineralization, while triggering the secretion of growth factors, i.e., IGF-I and IGF-II, which are fundamental for bone metabolism [36]. Moreover, it should be considered that additional amounts of Ca will result in a further increase in the Ca/P ratio in the glass composition, thus positively affecting the bioactive potential of the glass, likewise to what was observed in the first bioactive composition 45S5, characterized by a high Ca/P ratio and an exceptional bioactive potential [9]. Furthermore, there is experimental evidence proving that MgO has a positive effect on cell adhesion because of the beneficial interaction between integrins (cell membrane proteins) and magnesium ions [36].

**Figure 2:**
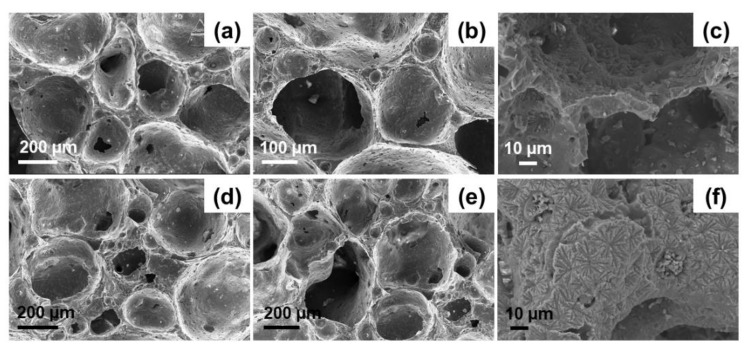
FE-SEM images of dolomite-foamed scaffolds D-800 (**a**–**c**) and D-850 (**d**–**f**) at different magnifications.

FE-SEM images of the cross-sectional fracture surfaces of D-800 and D-850 scaffolds are displayed in Figure 2. These morphological investigations show that both D-800 and D-850 scaffolds exhibited a quite homogeneous distribution of large macropores in their whole volume, without any apparent preferential orientation. This is consistent with the results reported by Fernandes et al. [35], who created isotropic porous interconnected structures from waste glass by using dolomite as a foaming agent. Small closed pores deriving from the foaming process can also be observed in the scaffold solid part (struts and walls), which is nonetheless well-densified.

Interestingly, the typical bubble-like architecture resulting from the dolomite-foaming method (Figure 2) exhibits a strong similarity to that of traditional sol–gel glass scaffolds which are foamed by using a surfactant [10,43,44]. In general, interconnected spheroidal pores ranging from 100 µm to 250 µm with inter-pore windows up to 50 µm can be observed in Figure 2. These porous biomaterials are therefore suitable for possible use in bone repair applications, for which scaffolds with a minimum pore size of 100 μm are typically recommended [45]. It is worth noting that the scaffolds sintered at the higher temperature (D-850) exhibited smaller inter-pore channels and lower inter-pore connectivity due to the higher apparent density and porosity reduction achieved by increasing the sintering temperature. This is confirmed by porosity evaluation through gravimetric method, which revealed a total pore content of 83.1 ± 2.2 vol.% and 65.5 ± 7.1 vol.% for D-800 and D-850 samples, respectively. In both cases, scaffold porosity still is comparable to the typical range of human trabecular bone (50–90 vol.%) [46].

Apart from the characteristic elements of the base glass composition (Mg, Ca, Si, Na, P, K, and O), EDS analysis revealed the presence of a non-negligible amount of carbon (>6 wt.%) in both D-800 and D-850 scaffolds, which might be attributed to an incomplete decomposition of dolomite upon thermal treatment.

Figure 2c,f, displaying the presence of crystalline phases, suggests that devitrification occurred upon sintering at both 800 °C and 850 °C. This is consistent with the results reported by Fiume et al. [47] in a previous work, where the sinter-crystallization of 47.5B glass was studied in detail. Specifically, DTA analysis carried out on 47.5B glass powder at heating rates ranging from 10 to 40 °C/min revealed the presence of an exothermic peak in the range of 765–848 °C [47]. As the onset crystallization temperature (T_x_) and peak crystallization temperature (T_p_) typically increase with the heating rate, it is reasonable to predict a shift of the position of T_x_and T_p_ towards lower temperatures due to the lower heating rate used in the present study for scaffold sintering (5 °C min^−1^), thus further justifying the nucleation of crystalline species on the surface of the materials.

**Figure 3:**
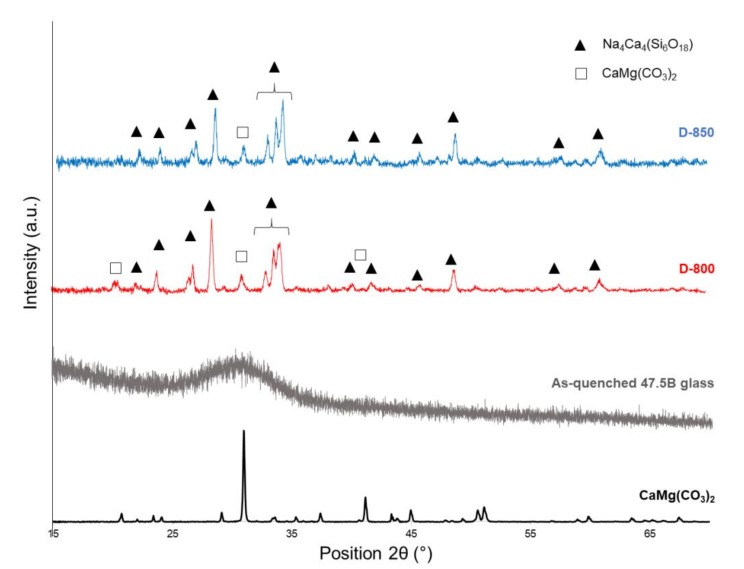
XRD patterns of pure dolomite (black), 47.5B glass (grey), D-800 (red) and D-850 (blue) powdered scaffolds.

XRD analyses performed on CaMg(CO_3_)_2_ powders, as-quenched 47.5B glass, D-800 and D-850 powdered scaffolds are shown in Figure 3.

Consistently with FE-SEM observations, XRD patterns referred to in D-800 and D-850 scaffolds showe the presence of diffraction peaks, associated with a single crystalline phase Na_4_Ca_4_(Si_6_O_18_) (ref. code: 01-079-1089), in agreement with what our previous study reported about bread-derived glass–ceramic scaffolds sintered at 750 °C [21]. Interestingly, the same sodium–calcium silicate phase (combeite-type phase) was also reported by other authors [48,49] to form above 550 °C in partially crystallized 45S5 Bioglass^®^, which is the common reference among bioactive glasses and glass–ceramics in terms of biocompatibility and bioactivity. On the other hand, the as-quenched 47.5B glass showed the typical XRD pattern of an amorphous material, characterized by a broad halo in the range 25°–35° (Figure 3). Furthermore, a direct comparison between the XRD pattern of dolomite (foaming agent) and those of D-800 and D-850 scaffolds allowed us to detect the presence of the main peak of CaMg(CO_3_)_2_ in the diffraction patterns of the sintered scaffolds at 2θ = 31.2°. The presence of residual dolomite explains the detection of carbon in the elemental analysis of scaffolds performed by EDS.

Full decomposition of dolomite could be achieved by applying a thermal treatment above 850 °C; however, it is strongly believed that sintering at higher temperatures might be an inconvenient strategy for the intended purpose. In fact, considering the properties that bioactive glass-based scaffolds for bone tissue engineering should have, the proper balance of good structural/morphological features, sintering, crystallization behavior, and hydroxyapatite (HA)-forming ability is considered a crucial point in order to guarantee good performances over the whole duration of the clinical treatment. Higher sintering temperatures, in fact, might cause an excessive increase in the density of the structure, which would become too high as compared to the reference range of human trabecular bone [50] reported in Table 2. As a consequence, porosity would be significantly reduced, too, if sintering is performed at higher temperatures. According to these considerations, T = 850 °C was deliberately selected as the maximum sintering temperature in this study. The biocompatibility of 47.5B-derived glass–ceramic materials was demonstrated elsewhere using fibroblasts [37]; further studies of cytocompatibility deserve to be performed on the foamed scaffolds that might contain small residual amounts of dolomite. In this regard, it is worth mentioning that the biocompatibility and osteogenic potential of dolomite were previously studied in a rat calvarial model by Moreschi et al. [51], who observed a moderate inflammatory response with no osteoconductive activity. However, despite this apparently negative result, the bone repair process appeared to be favored in the presence of dolomite compared to the negative dolomite-free control [51].

**Figure 4:**
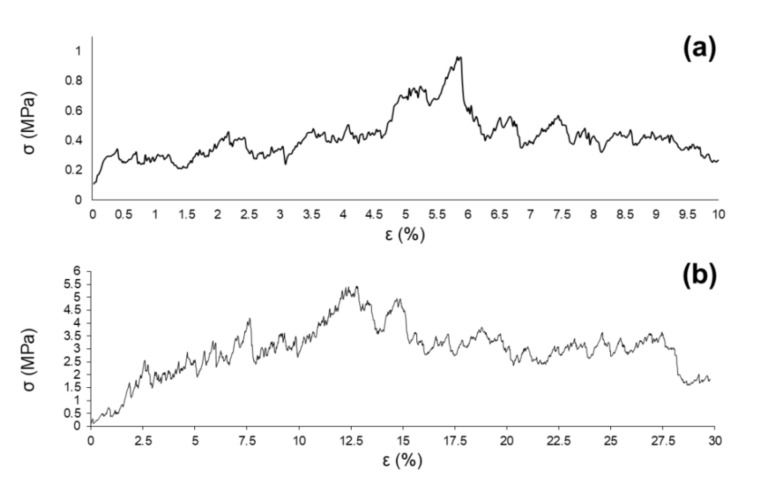
Stress–strain (σ-ε) curve of dolomite-foamed D-800 (**a**) and D-850 scaffolds (**b**).

Figure 4 shows a couple of examples of stress–strain curves related to D-800 (Figure 4a) and D-850 (Figure 4b) scaffolds.

In both cases, the curves are characterized by the typical trend of a cellular ceramic material, with several peaks associated to multiple fractures that occurred upon compression until collapse was achieved by brittle crushing, according to the Ashby’s model [52]. As a result of the compressive test, the scaffolds lost their structural integrity and were reduced in small fragments; the mechanical test was interrupted before starting the compression of fractured foams, which would have been associated with densification and misleading increase of the stress [52].

A summary of the structural features of dolomite-foamed scaffolds produced in this study compared to the typical reference ranges of human trabecular bone is provided in Table 2.

Apart from the previously discussed density levels, which are comparable to the reference values reported for human trabecular bone, both D-800 and D-850 scaffolds exhibited porosity and compressive strength values potentially suitable for bone tissue engineering applications. It is reasonable to attribute the better σ_c_ of D-850 scaffolds (3.9 ± 0.9 MPa) to the higher densification of the struts obtained upon sintering, resulting in a consequent decrease of the porosity of the foams. Despite the compressive tests performed on D-800 scaffolds yielding σ_c_ = 1.3 ± 0.4 MPa, which is one third that of D-850 scaffolds, this value is definitely above the lower limit of human trabecular bone (0.1–16 MPa [46]) and remarkably higher than the results reported for foam-replicated 45S5 Bioglass^®^ scaffolds (0.2–0.4 MPa) [16].

**Figure 5:**
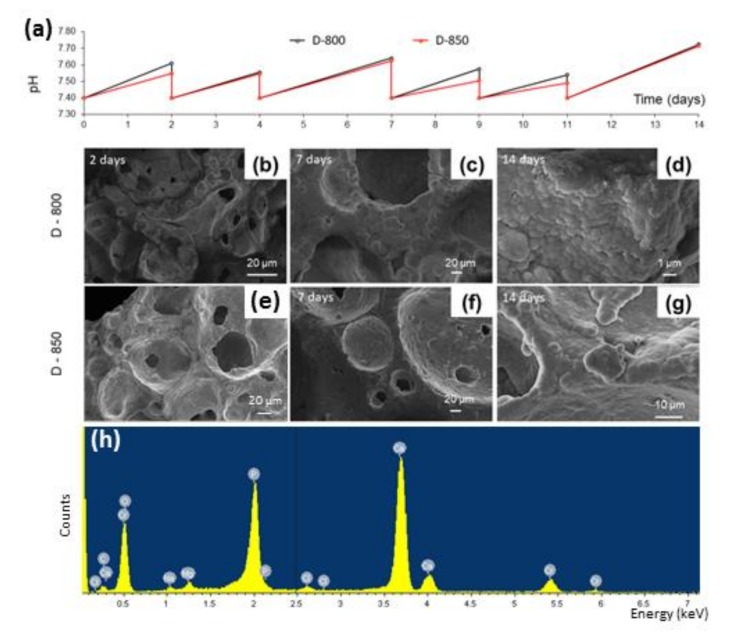
Bioactivity tests in simulated body fluid (SBF): pH variation as a function of the immersion time (**a**); FE-SEM micrographs of the surface of D-800 (**b**–**d**) and D-850 (**e**–**g**) scaffolds after immersion in SBF for 2, 7, and 14 days; example of EDS spectrum of the newly formed surface layer on D-850 sample after seven days in SBF (**h**).

As regards in vitro bioactivity tests, the variation of pH as a function of the soaking time in SBF is plotted in Figure 5a.

The curves obtained for D-800 and D-850 scaffolds are characterized by the same trend: all the ramps between each time point indicate the increase of pH associated with the release of ions from the surface of the scaffold to the solution, consistent with the in vitro bioactivity mechanism typical of silica-based bioactive glasses [53]. The drop of pH observed every 48–72 h, instead, was due to the complete replacement of the solution, which was performed to simulate fluid recirculation in physiological conditions.

No problems of pH-related toxicity induced by the materials were forecast, as the maximum value of pH reached upon soaking was around 7.7 for both scaffolds. Such a pH increase could even be beneficial to the activity of osteoblasts that are typically stimulated in a mildly alkaline environment [54].

In vitro bioactivity tests in SBF revealed that the scaffolds possessed a good HA-forming ability, which was not apparently affected by the high sintering temperatures and the devitrification of the material (foam). After just 48-h immersion, the surface appeared to be covered by a thin layer of HA, below which a cracked reaction layer of silica gel was clearly visible (Figure 5b,e). Small globular aggregates were observed also onto the inner surface of the pores, suggesting that the fluid properly permeated the whole volume of the 3D structure thanks to the open-cell architecture achievable by the manufacturing method here presented.

An increasing number of globular agglomerates were observed after seven-day immersion (Figure 5c,f) as proof of the continuous ion exchange between the glass and the solution, and the progress of the bioactivity mechanism over time.

After two weeks, the surface of the scaffolds appeared to be completely covered by a homogeneous layer of globular HA characterized by its typical cauliflower morphology (Figure 5d,g). Semi-quantitative EDS analyses of the scaffold surfaces after immersion in SBF for 14 days yielded Ca/P molar ratios of 1.60 ± 0.04 and 1.56 ± 0.06 (calculated on five sites) for D-800 and D-850 samples, respectively. These values are close to that of stoichiometric HA (1.67), thus indicating an advanced stage of the conversion reaction of the glass surface to HA, which might result in a better interaction between the scaffold and the host tissue due to the higher similarity to the calcium–phosphate mineral phase of bone. EDS analysis revealed that the Si peak (typical of the 47.5B-derived materials) disappeared just after one-week immersion in SBF, while the peaks of Ca and P are well visible (Figure 5h), thus indicating that the surface of the scaffolds was completely covered by a relatively thick calcium phosphate layer (the peaks of chromium are due to metal coating prior to the analysis, the small peaks of Na, Mg, and Cl can be associated to traces of salts formed during immersion).

Formation of an HA layer on the surface of biomaterials is commonly considered as a key requirement to allow interfacial bonding with bone after implantation in the living body as osteoblasts will attach and proliferate on this calcium–phosphate layer, producing new bone [55]. Figure 5 suggests that the pore interconnectivity still persisted during immersion in SBF, regardless of the growth of the HA layer that tends to slightly reduce the macropore size. This is consistent with previous studies on macroporous glass scaffolds reporting that the thickness of the surface HA layer can reach 20-30 μm, yet allowing scaffold permeation by bio-fluids and cells [56,57].

**Figure 6:**
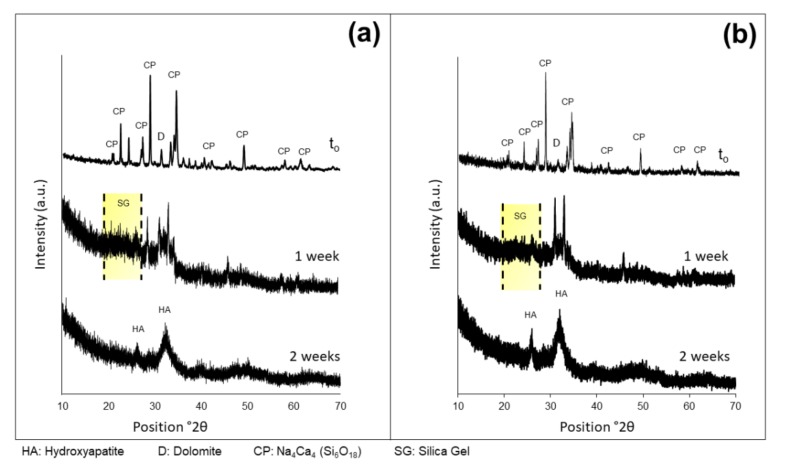
XRD patterns after immersion in SBF at different time points related to D-800 (**a**) and D-850 (**b**) scaffolds. CP = crystalline phase (Na_4_Ca_4_(Si_6_O_18_)) of sintered glass-ceramic scaffold; D = residual dolomite; SG = halo associated to the silica gel layer; HA = hydroxyapatite.

As a further proof of the good HA-forming ability of the scaffolds, XRD analyses were carried out on D-800 and D-850 samples immersed up to 7 and 14 days in SBF (Figure 6) and compared to the patterns of the starting materials.

The XRD patterns related to the two analyzed systems evolved following a comparable trend. The analysis revealed that the diffraction peaks identifying the scaffold crystalline phase were nearly no longer visible after one-week immersion in SBF, while a broad peak was detected at around 32° ((211) major reflection of HA) as well as the presence of a halo between 20°–30° associated to the formation of the reaction layer of silica gel. After two-week immersion in SBF, the major peak of HA became a bit shaper and a secondary peak appeared at 26.2°, corresponding to the (002) reflection of HA [58].

Collectively, the results of EDS, FE-SEM, and XRD analyses obtained from D-800/D-850 scaffolds after in vitro bioactivity tests suggested no significant influence of the sintering temperature on the HA-forming ability of samples, as HA deposition kinetics and morphological features were comparable in both systems analyzed.

## 4. Conclusions

In this work, dolomite foaming was implemented for the first time to obtain 3D porous bioactive glass-based scaffolds for medical applications in bone tissue engineering. This strategy represents an interesting and novel example of how waste material (e.g., dolomite powder from stone processing) could be used to fabricate high-added-value products for advanced applications like biomedical glass-derived foams. Morphological, structural, and bioactive properties of the obtained glass–ceramic scaffolds were found to be potentially suitable for the intended purpose. Increase of the sintering temperature from 800 °C to 850 °C led to a favorable increase in the mechanical properties of the structures under compressive loads and did not affect the HA-forming ability of the material in SBF. Further studies could be carried out to determine the influence of residual dolomite on cellular viability and metabolism. Taken together, these results are promising and open up new possibilities in the field of scaffold manufacturing for bone tissue applications.

## Figures and Tables

**Table 1:** Summary of physical properties of precursors used for the production of the foams.

Precursor (Powder)	Amount in the Scaffold (wt.%)	Particle Mean Size (µm)	Density (g/cm3)	Specific Surface Area (m2/g)
**47.5B Glass**	98	16.57	2.64	0.638
**Dolomite**	2	12.79	2.86	0.834

**Table 2:** Summary of major physical and structural parameters of dolomite-foamed scaffolds.

Sample	Apparent Density (g/cm^3^)	Porosity (vol.%)	Compressive Strength/σ_c_ (MPa)
Trabecular Bone	0.18–0.56 [50]	50–90 [46]	0.1–16.0 [46]
D-800	0.45 ± 0.050	83.1 ± 2.2	1.3 ± 0.4
D-850	0.91 ± 0.17	65.5 ± 7.1	3.9 ± 0.9
